# A life-threatening encounter: an uncommon case of ruptured hydatid cyst presenting as anaphylactic shock and respiratory distress in a 12-year-old boy

**DOI:** 10.1097/MS9.0000000000001330

**Published:** 2023-09-18

**Authors:** Abulfazl Vantankhah, Pegah Bahrami Taqanaki, Mohsen Rahmanian, Leila Ameri, Khashayar Atqiaee, Mahdi Parvizi Mashhadi

**Affiliations:** a School of Medicine, North Khorasan University of Medical Sciences, Bojnurd; b Department of pediatric surgery, Mashhad University of Medical Sciences; c Parsian Imaging Center; d Department of Pediatric Surgery, Mashhad University of Medical Sciences, Mashhad, Iran

**Keywords:** hydatid cyst, respiratory distress, loss of consciousness, case report

## Abstract

**Introduction and importance:**

Although rare, the spontaneous rupture of a lung hydatid cyst or its perforation into the pleural cavity can give rise to an abrupt onset of symptoms, including cough, fever, hemoptysis (coughing up blood), and hypersensitivity reactions, and can ultimately lead to respiratory failure.

**Case presentation:**

A 12-year-old boy was brought to the emergency room with a loss of consciousness. Symptoms included tachypnea, fever, low blood pressure, and overall respiratory distress. After resuscitation, a chest X-ray revealed a distinct, well-defined round opacity located in the lower region of the right lung, leading to mediastinal displacement. After confirmation of the disease, the child was hospitalized in the ICU care and consequently underwent surgery. Treatment was successful and there was no recurrence on the follow-up.

**Clinical discussion:**

Studies have demonstrated that the right lower lobe of the lung is the most frequently affected area of the lung by hydatid cysts. Symptomatic and complicated hydatid cysts are a rare concept in children, and only a small percentage, are diagnosed in patients younger than 16 years. Surgery remains the preferred treatment for the majority of patients with pulmonary hydatid disease. It is important to note that combined surgery and chemotherapy represents the current gold standard in managing pulmonary hydatid cyst.

**Conclusion:**

Although anaphylactic shock caused by a ruptured lung hydatid cyst is rare, it should be taken into consideration by physicians as a differential diagnosis in patients who also have respiratory symptoms, particularly in endemic areas.

## Introduction

Highlights

- Although rare, the spontaneous rupture of a lung hydatid cyst or its perforation into the pleural cavity can give rise to an abrupt onset of symptoms, including cough, fever, hemoptysis (coughing up blood), and hypersensitivity reactions, and can ultimately lead to respiratory failure.
- Symptomatic and complicated hydatid cysts are a rare concept in children, and only a small percentage, are diagnosed in patients younger than 16 years.
- Although anaphylactic shock caused by a ruptured lung hydatid cyst is rare, it should be taken into consideration by physicians as a differential diagnosis in patients who also have respiratory symptoms, particularly in endemic areas.

Cystic echinococcosis (CE) is an enduring cystic helminthic disease of zoonotic nature caused by the metacestode stage of the taeniid cestode *Echinococcus granulosus*. Within the conventional life cycle, *E. granulosus* tapeworms inhabit the intestinal tract of canines, primarily dogs, which serve as the definitive host. Upon ingestion of cysts present in infected viscera, protoscoleces adhere to the canine’s intestine and undergo maturation into fully developed adult tapeworms within ~40–45 days. Subsequently, the eggs of these tapeworms are excreted in the faeces, disseminated extensively, and are capable of enduring environmental conditions for a minimum of 1 year^1–3^. Upon being ingested by a suitable intermediate host, typically herbivorous animals such as sheep, cattle, goats, pigs, horses, or camels, the oncosphere (embryo) within the outer egg capsule undergoes release. The oncosphere passes through the duodenal mucosa and gains entry into a portal vein branch. Subsequently, it settles within various tissues, predominantly the liver and lungs, which serve as crucial filtering organs. Furthermore, flies and other arthropods can act as intermediaries in the transmission process, facilitating the potential infection of humans^1–4^. Humans can serve as unintentional intermediate hosts and contracting an infection through faecal-oral transmission, particularly during close and intimate interactions between children and dogs. As a consequence of this transmission, individuals may develop substantial cysts several years after the initial infection^2,5^. These cysts, known as echinococcal cysts or metacestodes, are characterized by their fluid-filled, spherical structure. Within the inner layer of the cyst, scolices, which are the infectious embryonic tapeworms, develop from an outpouching called the brood capsule. The cyst fluid is described as crystal clear and is a transudate of serum-containing proteins. If the cyst fluid is released into the host’s bloodstream, it can result in eosinophilia or anaphylaxis, although cyst rupture may not present noticeable clinical symptoms^2,4^. In human cases, the hydatid cysts (HC) grow slowly over time, eventually reaching sizes of several liters and containing numerous protoscolices^2^.

The prevalence of hydatidosis persists in various regions across the globe, including temperate countries like the Mediterranean region, Australia, New Zealand, southern and central areas of the former Soviet Union, Central Asia, the Middle East, China, South America, and certain parts of Africa^1,3,6,7^. The majority of cases in the USA and Central Europe occur in immigrants from endemic areas.

Various factors have been identified as risk factors for human CE, including engagement in pastoral occupations, a history of dog ownership, lower levels of education, age, sex, and the source of drinking water^2,3^.

In adults, the liver is the primary site of involvement, while in children, the lungs are predominantly affected by CE. Lung cysts typically develop through the hematogenous dissemination of larvae via the hepatic sinusoids. The spontaneous rupture of a lung cyst or its perforation into the pleural cavity can give rise to an abrupt onset of symptoms, including cough, fever, hemoptysis (coughing up blood), and hypersensitivity reactions, and can ultimately lead to respiratory failure^2^. This paper introduces a rare case of a ruptured lung hydatid cyst presenting as anaphylactic shock and respiratory distress.

## Case presentation

A 12-year-old boy with no known chronic illness or history of medical conditions, from a rural region in the countryside of Bojnurd city, was brought to the emergency room (ER) with a decreased level of consciousness. Based on the history provided by the parents, the boy experienced unconsciousness for ~30 min before being taken to the ER. The parents did not mention any tonic movements, foaming at the mouth, or cyanosis occurring at home.

Upon arrival at the ER, the patient’s condition was characterized by tachypnea with a respiratory rate of 47 breaths per minute which was concurrent with probable signs of respiratory failure including belly breathing, noisy respiration along with intercostal and suprasternal retractions.

The patient’s vital signs were a blood pressure of 95/50 mmHg, oxygen saturation of 83%, and an axillary temperature of 38.3°C. Physical examination reveals crackles on lung auscultation, which are concurrent with a unilateral reduction of respiratory sounds at the lower zone of the lung. These findings are more pronounced in the right lung. Additionally, the examination of the mouth shows large tonsils. The patient did not respond to verbal stimulation and only demonstrated withdrawal in response to pain stimuli. Pupillary examination showed normal findings. In the physical examination, there were no signs of skin rash, insect bite, head trauma, or biting of the tongue. The arterial blood gas examination revealed respiratory acidosis (PH: 7.21, PCO_2_: 73, and HCO_3_: 28.7).

After attending Airway, Breathing, and Circulation in resuscitation primary surveys, which included protection of the airway, oxygenation, IV line access, and administration of isotonic IV fluids, the patient received intravenous hydrocortisone, a short-acting beta-agonist nebulizer, and intravenous phenytoin. As a result of this treatment, the patient’s consciousness was regained within 10–15 min.

After regaining consciousness, the patient reported that in the morning, he woke up from sleep and had several severe and spontaneous coughs, and he does not remember what happened afterward. He stated that he experienced a flu-like illness in the past several days and did not take any medication for this flu-like illness. The patient did not have symptoms of progressive dyspnoea, consumption of a new drug or food, previous history of seizure and epilepsy, or any other relevant symptoms that could lead to a diagnosis. There was also no significant familial medical history reported by the parents.

Laboratory examinations showed a leucocyte count of 20 300/mm^3^ (consisting of 47% neutrophil, 47% Lymphocyte, 3% Monocyte, and 3% Eosinophil, indicating leukocytosis and eosinophilia), Haemoglobin of 12.7 g/dl, Platelet count of 376 000/μl, and C-reactive protein level of 35 mg/l. All other laboratory examinations were within the normal range.

Based on the initial presentation and symptoms, the major diagnosis was a seizure, and it was assumed that the child was in the post-ictal phase. Symptoms like tachypnea and lung crackles were suspected to be related to aspiration during a seizure attack. Aspiration during a seizure can lead to respiratory complications, which may explain the tachypnea and lung crackles observed during the physical examination. Alternative differential diagnosis, ranked in order of priority encompassed asthma exacerbation, anaphylactic reaction, foreign body aspiration, central nervous system infections, and pneumonia.

**Figure 1:**
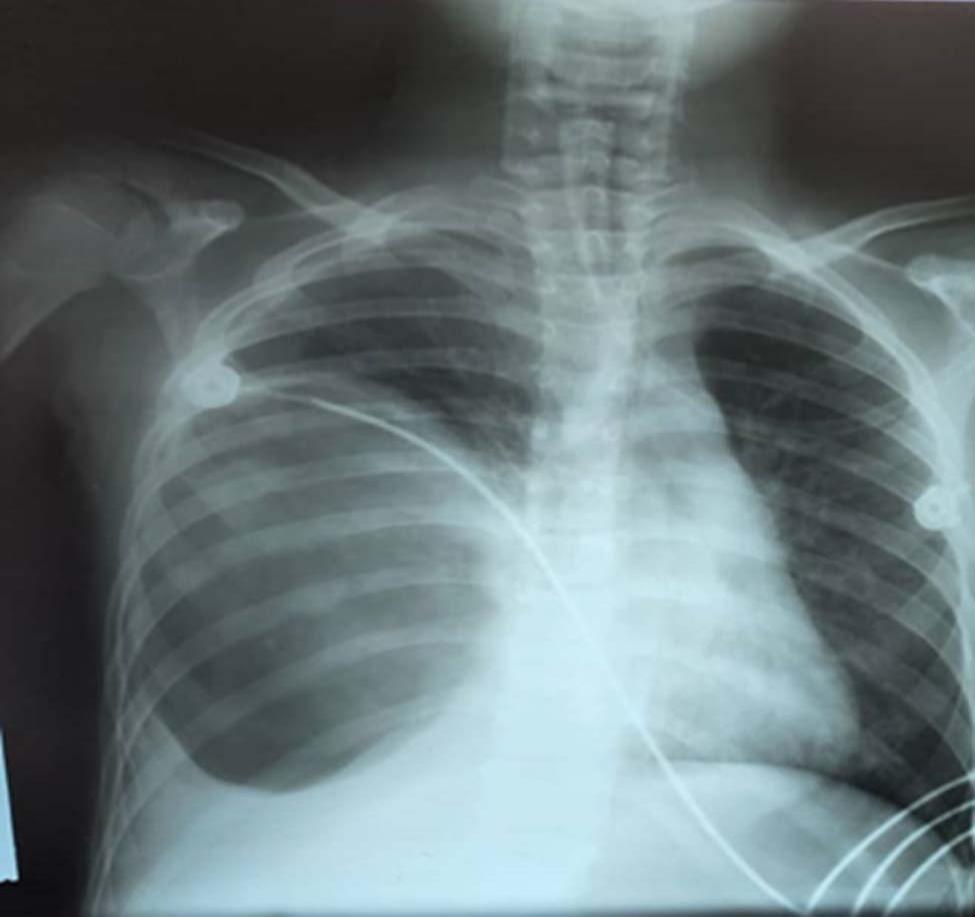
Depicts a chest X-ray image revealing a distinctive round, sharp-edged opacity situated in the lower region of the right lung, as highlighted by white arrows.

To eliminate the aforementioned range of potential diagnoses, an anteroposterior chest X-ray was performed in the ER, revealing a distinct, well-defined round opacity located in the lower region of the right lung, leading to mediastinal displacement (Fig. 1). The most plausible differential considerations for this particularly rounded opacity encompassed hydatid cystic disease, diaphragmatic eventration, and both benign and malignant lung neoplasms.

**Figure 2:**
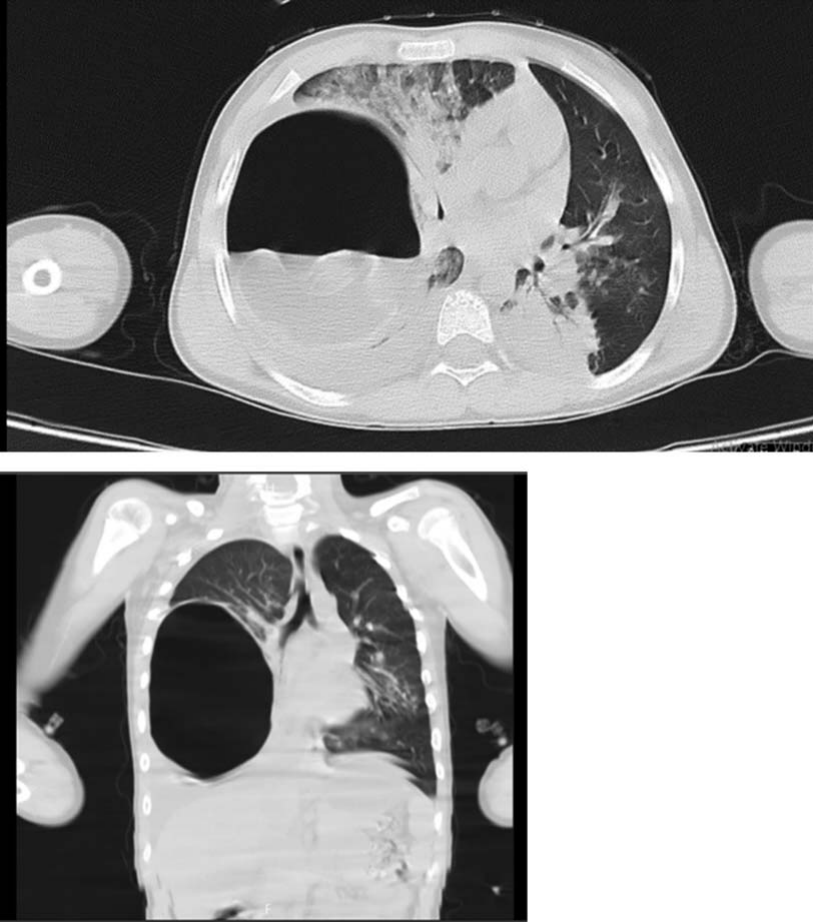
Shows a prominently large ruptured cyst, measuring ~132×125×97 mm, in the right hemi thorax. The cyst exhibits an air-fluid level and a floating membrane, indicative of its ruptured state. Additionally, moderate pleural effusion is observed, accompanied by a patchy ground glass appearance and scattered consolidations in the thorax’s right and left lower zones.

Upon the patient’s stabilization, a computed tomography scan was conducted, revealing a ruptured cyst exhibiting a water lily sign in the lower region of the right lung. This characteristic imaging appearance is indicative of a ruptured HC (Fig. 2).

Following the confirmation of the diagnosis, the patient was promptly admitted to the paediatric intensive care unit in preparation for surgical intervention. Following the patient’s thoracotomy, the surgical team proceeded with cystectomy and capitonage. However, due to the significant extent of lung involvement, the decision was made to perform a lobectomy of the inferior right lobe. To allow for the expulsion of residual air and fluid, a chest tube was inserted post-surgery. Subsequent to the surgical intervention, the patient underwent a 3-month course of albendazole treatment, which proved efficacious in preventing recurrence. Follow-up assessments exhibited no indications or symptoms of disease relapse.

## Discussion

CE in humans is characterized by the formation and enlargement of fluid-filled cysts primarily in the liver and lungs. However, it is important to note that the disease can also affect other anatomical sites such as the abdominal cavity, heart, bone, muscle, nervous system, and other locations throughout the body^3^. In children, the lung is the predominant site for cyst detection, apparently attributable to its compressible nature, vascularization, and negative pressure. The lung facilitates cyst growth through negative pressure and great elasticity, resulting in a faster growth rate compared to the liver by a ratio of 3:1^1,8^. Pulmonary lesions can manifest as either primary or secondary occurrences resulting from the intrathoracic rupture of a hepatic HC. The size of pulmonary cysts can vary within the range of 1–20 cm in diameter. In general, pulmonary HCs tend to remain asymptomatic until they experience rupture. The clinical presentation observed in patients is directly influenced by whether the cyst is intact or has undergone rupture^6^.

The lower right lobe shows the highest frequency of lung involvement, with ~60% of cases observed in the lower lobes. Bilateral involvement occurs in 20% of cases and about 30% of cases present with multiple cysts.

Most pulmonary cysts are primary cysts, which are formed as a filled cavity and composed of three distinct layers. The first layer is the pericyst, which originates from the host and consists of compressed lung tissue accompanied by an inflammatory host reaction elicited by the parasite, leading to fibrosis. The second layer is the ectocyst, also known as the laminated layer or hyaline membrane. Finally, the third layer is the endocyst, referred to as the germinal layer^1^. In the case of a simple cyst, the pulmonary tissue surrounding the adventitia may undergo compression, leading to atelectasis, or exhibit irreversible inflammatory alterations such as bronchiectasis and interstitial sclerosis^8^. When a cyst ruptures, whether due to spontaneous trauma or medical intervention, the abrupt discharge of its contents can trigger allergic reactions, varying in severity from mild to potentially life-threatening anaphylaxis^1^. In the lungs, if the cyst membranes rupture, their contents can be expelled through the bronchi, resulting in the expectoration of cyst fluid, membranes, and scolices. Alternatively, the ruptured membranes may be retained within the lungs, providing a site for potential bacterial or fungal infection. Moreover, protoscolices present in the cyst fluid can disseminate, giving rise to secondary cysts in the nearby tissues and contributing to allergic and anaphylactic reactions. The expectoration of cystic contents can give rise to severe complications, including acute respiratory failure, massive hemoptysis, and anaphylactic shock^6,8^.

Typically, there is no universally accepted size criterion to categorize an HC as a “giant.” However, cysts with a diameter surpassing 10 cm are commonly considered giant hydatid lung cysts. Giant HCs are more frequently found in children who are older than 10 years^9,10^.

The most commonly reported symptoms include cough (53–62%), often paroxysmal in nature, chest pain (49–91%), dyspnoea (10-70%), hemoptysis (12–21%), fever (15%), and purulent sputum (12%)^1,10,11^. Dyspnoea and thoracic pain were notably more frequently reported in cases involving giant cysts^12^. Less frequently described symptoms consist of dyspnoea, malaise, nausea, vomiting, and thoracic deformations. A large proportion of children and adolescents with lung lesions exhibit no symptoms, even though they may have sizable lesions. This is presumed to be due to a weaker immune response and the relatively higher elasticity of the lung parenchyma in this age group. In numerous cases, infections are asymptomatic and are incidentally detected during echographic screenings or postmortem examinations (autopsy)^1,13^.

In our case the child presented as hemodynamically unstable with loss of consciousness and severe respiratory distress which was first misdiagnosed as seizure related aspiration. This gives rise to the important issue of having hydatidosis in mind as a differential diagnosis in endemic areas.

Isolated pulmonary cysts are more prevalent in children compared to adults. It is noteworthy that a significant proportion, ranging from 20 to 40% of individuals, may have multiple cysts or involvement of multiple organs in cases of hydatid infections. Notwithstanding hydatid infections may be acquired during childhood, it is noteworthy that most cases involving liver and lung cysts become symptomatic and are diagnosed in adulthood. This is mainly attributed to the slow growth rate of the echinococcal cysts. Only a small percentage, around 10–20% of cases, are diagnosed in patients younger than 16 years^2,14^.

As the HC grows, it causes erosions in the bronchioles that are surrounded by the pericyst. This leads to the introduction of air between the pericyst and the laminated membrane. This air collection appears as a thin, radiolucent crescent in the upper part of the cyst, which is known as the crescent sign or meniscus sign. Following the partial expectoration of the cyst fluid and scolices, the cyst starts to empty, and the collapsed membranes become visible inside the cyst, resembling a serpent sign. As the cyst fully collapses, the crumpled endocyst can be seen floating freely in the cyst fluid, resembling a water lily sign^11^.

The local complications of pulmonary hydatidosis can be grouped as follows: rupture into the parenchyma and pleural cavity, secondary infection (cyst abscess), anaphylactic reaction, reactions of adjacent tissues, and complications resulting from space occupation^4,15,16^. Rupture represents the predominant complication of pulmonary hydatid disease (HD), with a reported incidence of 49%.

Approximately 10–20% of cases of HC disease are diagnosed during childhood. In individuals with a history of exposure to sheepdogs in regions where Echinococcus granulosus is endemic, the presence of a cyst-like mass strongly supports the diagnosis of CE^2,17^. The diagnosis of human echinococcosis heavily relies on imaging techniques such as computed tomography scan, MRI, ultrasound, and radiography. Laboratory-based diagnosis, such as antibody assays, serves as a valuable tool to confirm presumptive radiologic diagnoses. This methods mainly rely on detecting specific serum antibodies in suspected cases or people enroled in mass screening programs. However, it’s important to note that serological methods are never 100% sensitive and specific. As a result, some patients with CE may not produce a marked antibody response^3^. Irrespective of the location, the sensitivity of serologic tests exhibit an inverse correlation with the extent to which echinococcal antigens are sequestered within cysts. Specifically, intact and healthy cysts tend to provoke a response that is minimally detectable, while ruptured or leaking cysts are associated with more robust responses^2^. The differential diagnosis of pulmonary HD encompasses various conditions, including benign tumours, inflammatory masses, metastasis, solid or fluid-filled cysts, and carcinomas^18^.

Treatment of human hydatidosis proves challenging due to the predominant development of cysts or cystic lesions in vital organs such as the liver, lungs, or other anatomical sites^3^. Due to the considerable variability observed in pulmonary echinococcosis, the implementation of a standardized treatment regimen or approach may prove challenging. The absence of comprehensive clinical trials comparing all diverse treatment modalities further contributes to this complexity. Management decisions are influenced by various factors, including cyst size, characteristics, location within the lung, clinical presentation, as well as the accessibility of medical and surgical expertise and equipment^19^. Surgery remains the preferred treatment for the majority of patients with pulmonary HD^19^; however, it is important to note that combined surgery and chemotherapy represent the current gold standard in managing pulmonary CE. Albendazole, mebendazole, and praziquantel demonstrate cure rates of ~30% when utilized as standalone chemotherapy for the treatment of HD. For optimal outcomes, surgical excision of the cysts should be complemented with chemotherapy, employing albendazole at a dosage of 12–15 mg/kg/day for 28 days, or mebendazole at a dosage of 200 mg/kg/day in three divided doses for 16 weeks. Preoperative chemotherapy with albendazole or mebendazole is recommended to reduce the risk of secondary echinococcosis after the operation. Initiation of this chemotherapy should begin at least 4 days before the surgical procedure and continue for a duration of at least 1–3 months^3,20,21^.

The primary objective of surgery in the treatment of HD is the complete removal of the cyst, including the endocyst while ensuring closure of the pericystic cavity to prevent prolonged air leakage and empyema. Preservation of healthy lung parenchyma is also crucial, along with proper management of the residual cavity to prevent any potential adverse consequences associated with the spillage of cyst contents^2,19,22^. The choice of surgical approach in HD is influenced by several factors, including cyst size, whether it is intact or complicated, its unilateral or bilateral nature, whether it is solitary or multiple, and the extent of lung parenchyma destruction. Among the various surgical techniques, cystotomy and capitonnage are the most commonly applied methods in clinical practice^23^. In cases where an intact cyst ruptures, prompt surgical intervention becomes imperative to minimize the risk of infection, as the cyst cavity is now in communication with the airway, and the remaining membrane remnants can serve as a nidus for infection. Lobectomy is considered appropriate only in cases where the size and number of cysts and the extent of infection preclude less extensive procedures. The main indications for lobectomy include large cysts that affect more than 50% of the lobe, cysts with significant pulmonary suppuration that do not respond to preoperative treatment, multiple unilocular cysts, and complications arising from HD, such as pulmonary fibrosis, bronchiectasis, or severe haemorrhage. According to Vasquez and colleagues, hemoptysis could be indicative of advanced disease in hydatidosis. They observed that patients experiencing severe hemoptysis had a higher likelihood of requiring lung resection procedures, such as lobectomy or pneumonectomy. This association is likely due to the more severe damage to the lung parenchyma in these cases^24^. While efforts are made to preserve lung parenchyma through procedures like cystotomy and capitonnage, formal lung resection becomes necessary for treating advanced forms of HD^24,25^. In the study conducted by Arroud and colleagues, the criteria for lung resection were giant cysts that affected the entire lobe, extensive destruction of the lung parenchyma, or the presence of a pulmonary abscess. The researchers emphasized that the insidious progression of the cyst in children results in considerable and extensive destruction of the lung parenchyma^10^. Parenchymal resection was found to be more frequently performed in cases involving complicated cysts and giant cysts^10^. In the study conducted by Dincer *et al*.^25^, the criteria for lung resection included the presence of giant cysts that occupied the entire lobe parenchyma, extensive lung destruction, unresolved atelectasis following standard surgical procedures, the presence of abscesses, or severe pulmonary changes.

For pulmonary HCs, thoracotomy has been a well-established and safe surgical approach for an extended period. It enables the resection of the cyst and closure of broncho-cystic fistulas. Median sternotomy is a beneficial surgical approach for the treatment of bilateral anterior HCs. Thoracoscopy is a less invasive option but can be more challenging when dealing with fistulas. To prevent the persistence of a fistula, obliteration of the residual cavity (Capitonage) is recommended^20,22^. According to the findings of Anadol and colleagues, pulmonary cysts with a diameter of less than 9 cm showed a better response rate (72.0%) compared to cysts with a diameter of 9 cm or more (31.6%), and this difference was statistically significant. They concluded that medical therapy is the preferred treatment for childhood hydatidosis, except in cases of large pulmonary cysts or when complications such as infection and compression of lung parenchyma have occurred^26^. According to Kabiri and colleagues, in some patients with a large pleural effusion and severe pleural empyema, a temporary chest tube can be inserted followed by medical treatment. A few days later, when the patient is stable, thoracotomy is performed. Major resections may be necessary in cases of complicated empyemas with fibrothorax or huge cysts with bronchopleural fistulas. It is essential to note that delaying surgery for 10 days or more is considered a predictive factor for developing postoperative bronchopleural fistula and/or empyema^17^.

Surgery is not recommended for cysts that involve multiple organs or tissues or are located in high-risk areas^20^. The immediate postoperative complications following surgery for HCs include hemothorax, pneumothorax, and pyothorax^17^. The common surgical complications in children with pulmonary HCs include atelectasis, hydropneumothorax, wound infection, pleural reaction, and haemothorax. Additionally, other potential complications may develop, such as abscess formation, empyema, septic shock, bronchial rupture, pleural fistula, bronchiectasis, anaphylactic shock, and even mortality^27^.

Antiparasitic drugs are valuable in three instances: (1) for single, small, uncomplicated cysts, (2) in disseminated diseases, and (3) for patients with poor surgical risk^22^. Documentation of experience with chemotherapy using benzimidazole compounds is now extensive, and this medical approach can be recommended for many patients. Approximately a third of patients treated with benzimidazole drugs have been cured, and even higher proportions (ranging from 30 to 50%) have demonstrated significant regression of cyst size and alleviation of symptoms^2^. Mebendazole and albendazole share the same mechanism of action. However, albendazole exhibits a superior pharmacokinetic profile, leading to higher plasma concentrations compared to mebendazole. As a result, albendazole is more effective in young patients and in cases where the cysts are located in the lung or are small, uncomplicated, and solitary. For patients with non-complicated cysts and no contraindications to chemotherapy, a cycle of albendazole treatment can be suggested as the first-choice treatment^27–29^. As indicated by Kabiri *et al*.^17^, systemic adjuvant medical treatment is considered necessary as a routine approach. In severe disseminated cases or non-operable patients, the adjuvant medical treatment must be used for a more extended period of time.

The objective response to treatment is most effectively assessed by repeatedly evaluating cyst size and consistency at 3-month intervals using ultrasonography, computed tomography, or magnetic resonance imaging. Due to the highly variable time of recurrence, continuous monitoring of cyst size and consistency should be extended for at least 3 years^2^. Our study due to its nature as a case report hold the limitation of low generalizability by low number of cases.

## Conclusion

Although anaphylactic shock caused by a ruptured lung hydatid cyst is rare, it should be taken into consideration by physicians as a differential diagnosis in patients who also have respiratory symptoms, particularly in endemic areas.

## Ethical approval

It is exempt at our institution. Our institution does not give ethics approval reference numbers for case reports. Since this was a clinical case and it was managed clinically after the patient’s admission, ethics approval is not applicable.

## Consent

Written informed consent was obtained from the patient for publication of this case report and accompanying images. A copy of the written consent is available for review by the Editor-in-Chief of this journal on request”.

## Sources of funding

This research did not receive any specific grant from funding agencies in the public, commercial, or not-for-profit sectors

## Conflicts of interest disclosure

None.

## Author contribution

Study concept or design: M.P.M., A.V. Data collection: A.V., P.B.T., M.R. Supervision: M.P.M. Data analysis or interpretation: A.V., P.B.T., M.R., L.A., K.A., M.P.M. Writing the paper: A.V., P.B.T., M.R., L.A., K.A. Revision: M.P.M., A.V.

## Research registration unique identifying number (UIN)

None.

## Guarantor

Mahdi Parvizi Mashhadi.

## Provenance and peer review

Not commissioned, externally peer-reviewed.

## Data availability statement

All the required data are available in the manuscript.

## Acknowledgements

I thank my colleagues and co-authors for their expertise and assistance throughout all aspects of our study and for their help in writing the manuscript.
